# Neutrophil and neutrophil extracellular traps in acute kidney injury: from mechanisms to treatments

**DOI:** 10.3389/fimmu.2025.1688207

**Published:** 2025-10-15

**Authors:** Yifei Fu, Wenjuan Wang, Na Gong, Xumin Zheng, Xinru Guo, Kaiting Zhuang, Qiuxia Han, Zhe Feng, Xiangmei Chen, Guangyan Cai

**Affiliations:** ^1^ Medical School of Chinese PLA, Beijing, China; ^2^ Department of Nephrology, First Medical Center of Chinese PLA General Hospital, State Key Laboratory of Kidney Diseases, National Clinical Research Center for Kidney Diseases, Beijing Key Laboratory of Medical Devices and Integrated Traditional Chinese and Western Drug Development for Severe Kidney Diseases, Beijing Key Laboratory of Digital Intelligent TCM for the Prevention and Treatment of Pan-vascular Diseases, Key Disciplines of National Administration of Traditional Chinese Medicine, Innovation Team and Talents Cultivation Program of National Administration of Traditional Chinese Medicine, Beijing, China; ^3^ Department of Nephrology, Beijing Chao-Yang Hospital, Capital Medical University, Beijing, China

**Keywords:** acute kidney injury, neutrophil, neutrophil extracellular traps, peptidylarginine deiminase 4, inflammatory cascade

## Abstract

Immune-inflammatory dysregulation characterizes acute kidney injury (AKI) throughout its early progression and chronic evolution. Neutrophils and the neutrophil extracellular traps (NETs) they release play multiple roles in this process. Recent research indicates that NETs, characterized by their unique “DNA-histone-granule proteins (e.g., neutrophil elastase [NE], myeloperoxidase [MPO], proteinase 3 [PR3], and cathepsin G).” structure, have become a pivotal research focus in neutrophil biology, while their formation is intricately linked to signals within the tissue microenvironment. This review traces neutrophil dynamics from bone marrow development and recruitment to the kidney, culminating in suicidal or vital NETosis. It specifically compares neutrophil extracellular trap (NET) mechanisms in sterile versus infectious AKI. Besides, it details how non-specific NET components, while aiding pathogen and necrotic tissue clearance, simultaneously damage renal tubular epithelial and endothelial cells, amplifying inflammatory cascades. Furthermore, the review comprehensively summarizes therapeutic strategies targeting NETs for AKI, including inhibition of NET formation/release, blockade of specific NET components, and promotion of NET clearance. These studies offer new perspectives on the spatiotemporal-specific roles of NETs in AKI, laying a solid theoretical groundwork for advancing their exploration in AKI subtyping and precision therapy.

## 1 Introduction

Acute kidney injury (AKI) is a common and critical condition that poses a serious threat to human health. The global incidence of AKI varies widely, ranging from 0.7% to 31%, with an incidence of 19.4% in East Asia. In China, the estimated number of hospitalized AKI patients in 2013 was between 1.4 and 2.9 million, accounting for approximately 10% of national healthcare expenditures (1). However, as the detailed mechanisms underlying the onset and progression of AKI are not yet fully elucidated, effective interventions remain limited. This lack of understanding significantly increases the risk of progression to severe AKI and chronic kidney disease (CKD), resulting in substantial healthcare costs and a heavy societal burden (1, 2).The etiology of AKI is diverse, encompassing ischemic insult, drug-induced toxicity, rhabdomyolysis, and sepsis secondary to infection. Although the initiating factors vary, they converge on common pathophysiological mechanisms involving renal microvascular endothelial cells and renal tubular epithelial cells (RTECs) injury, immune dysregulation, oxidative stress, and microvascular dysfunction (3). Growing evidence indicates that the innate immune system, especially neutrophils, plays a critical and complex role in the initiation, progression, and repair phases of AKI.

As the most abundant and rapidly mobilized immune cells in the circulation, neutrophils contribute to inflammatory processes through phagocytosis, degranulation, and the formation of NETs. First described in 2004, NETs are extracellular web-like structures composed of decondensed chromatin and antimicrobial proteins, released via a distinct cell death pathway termed NETosis (4). Initially identified as a host defense mechanism against pathogens, NETs have since been implicated in a range of pathological conditions, exhibiting context-dependent beneficial and detrimental effects. In renal diseases, NETs contribute to cytotoxicity, complement activation, and thrombotic processes (5–8). However, owing to technical and conceptual challenges, research into neutrophil heterogeneity and the functional versatility of NETs remains limited. Current understanding of the formation, composition, and function of NETs within diverse immuno-microenvironments is still evolving.

Targeting NETs has emerged as a promising yet contentious therapeutic strategy. While inhibition of NET formation (9) or enhancement of their clearance (10) can attenuate tissue damage and excessive inflammation, such approaches risk compromising innate antimicrobial defenses, highlighting the delicate balance between NET-mediated protection and pathology. This duality underscores the need for deeper mechanistic insights and translational studies to elucidate the context-specific roles of NETs. At present, NETosis-directed therapies remain largely experimental and have not yet been validated in clinical AKI settings.

Therefore, this review aims to synthesize current evidence on the roles of NETs in AKI across various etiologies, summarize the signaling pathways governing NET formation and function under different pathophysiological conditions, evaluate recent advances in NET-targeting therapies, and identify key knowledge gaps and future research directions in the field.

## 2 Development and release of neutrophils in early-stage AKI

Neutrophils, as the most abundant innate immune cells in the body, play a pivotal role in rapidly initiating the inflammatory response during the early phase of AKI. Clinical studies have demonstrated significantly elevated neutrophil counts in the blood of AKI patients compared to non-AKI individuals (11). Furthermore, in various murine AKI models induced by different methods, increased infiltration of neutrophils in the renal tissue microenvironment has been consistently observed (12–14). Research confirms that significant neutrophil infiltration into renal tissues commences as early as 3 hours post ischemia-reperfusion injury (IRI) (15).

Specifically, neutrophils differentiate from multipotent granulocyte-monocyte progenitors derived from myeloid precursors, with their production under the coordinated control of the bone marrow microenvironment and systemic signals (16). Bone marrow neutrophils are categorized into three distinct developmental pools: the stem cell pool, the mitotic pool, and the post-mitotic pool (16). Upon full maturation within the post-mitotic pool, neutrophils possess complete granule systems and chemotactic receptors, and are released into neutrophil reservoirs or the marginated pool to serve as a functional bone marrow reserve (17, 18). Under homeostatic conditions, the high-affinity binding of C-X-C chemokine receptor type 4 (CXCR4) to C-X-C motif chemokine ligand 12 (CXCL12) anchors mature neutrophils within the bone marrow stroma. Conversely, during inflammation, granulocyte colony-stimulating factor (G-CSF) promotes the rapid egress of neutrophils from the bone marrow “marginated pool” into the circulation by downregulating CXCR4 expression and upregulating C-X-C chemokine receptor type 2 (CXCR2) (17, 18). This “on-demand release” mechanism endows neutrophils with the capacity for a swift response to tissue damage in the early stages of AKI.

## 3 Recruitment and migration of neutrophils during early AKI

To eliminate pathogens, damaged tissues, and promote repair, the renal tissue microenvironment post-AKI releases adhesion molecules and chemokines to recruit neutrophils. A study employing standardized ligand-receptor scoring for quantification revealed a specific temporal progression in cellular communication between renal cells and leukocytes during injury repair: endothelial cells initiate signaling to leukocytes first, followed sequentially by leukocytes themselves and injured renal tubular cells (19). Therefore, this review will primarily elucidate the recruitment of neutrophils by different injured cell types within the AKI microenvironment from the perspective of intercellular interactions.

### 3.1 Recruitment of neutrophils by renal vascular endothelial cells

Renal vascular endothelial cells constitute the critical gateway for circulating immune cells to infiltrate tissues and serve as a pivotal initiating component in the inflammatory cascade. Under physiological conditions, endothelial cells maintain barrier integrity through tight junction proteins (e.g., occludin, claudin) and adherens junction proteins (e.g., VE-cadherin); however, upon stimulation by inflammatory cytokines or pathogenic toxins, endothelial cells undergo cytoskeletal reorganization, leading to phosphorylation-dependent degradation or internalization of junctional proteins and the formation of paracellular gaps (20). These gaps provide direct physical channels for neutrophil transendothelial migration (TEM). Studies demonstrate that transgenic mice carrying a tyrosine-731-to-phenylalanine mutation (Y731F) in VE-cadherin (VEC-Y731F) specifically prevent the opening of endothelial junctions, significantly inhibiting neutrophil recruitment from the vasculature to perivascular tissues without altering vascular permeability (21). This confirms the essential role of endothelial junctional proteins in neutrophil TEM. Furthermore, migrating neutrophils release reactive oxygen species (ROS), proteolytic granules, and cytokines (e.g., directly degrading VE-cadherin), thereby widening endothelial gaps and establishing a positive feedback loop of “increased permeability-enhanced migration”. For instance, our previous research in ischemia-reperfusion injury induced AKI (IRI-AKI) models revealed that neutrophils highly express myeloid-related proteins MRP8/S100A8 and MRP14/S100A9, which further promote TEM of phagocytes (including neutrophils) by regulating cytoskeletal metabolism via microtubule reorganization (22, 23).

Moreover, following renal vascular endothelial injury, upregulated E-selectin, P-selectin, Intercellular adhesion molecule 1 (ICAM-1), and dipeptidase-1 (DPEP1) cooperate with corresponding integrins (including LFA-1 and Mac-1) to mediate neutrophil-endothelial adhesion post-AKI (24–27)。 Notably, the transient surge in plasma hepatocyte growth factor (HGF) after IRI activates the c-Met receptor on renal vascular endothelial cells, suppressing the NF-κB/ICAM-1 signaling pathway (28). This establishes a barrier effect that blocks neutrophil extravasation, representing a critical endogenous protective mechanism against renal IRI. Concurrently, chemokines bound to endothelial glycosaminoglycans (GAGs) form a solid-phase haptotactic gradient, providing directional migration cues for circulating neutrophils. Jens Bedke’s team demonstrated that dnCXCL8, a novel antagonist derived from human C-X-C motif chemokine ligand 8 (CXCL8) with high GAGs-binding affinity but low C-X-C chemokine receptor type 1/2 activation capacity, significantly inhibits endothelium-dependent neutrophil infiltration in AKI. (29, 30).

In summary, endothelial cells coordinately drive early neutrophil infiltration into renal tissues during AKI through dual mechanisms: disruption of physical barriers and regulation of chemical signaling, thereby exacerbating inflammatory injury.

### 3.2 Recruitment of neutrophils by renal tubular epithelial cells

RTECs, as essential parenchymal cells in renal tissue, dynamically interact with neutrophils in response to inflammatory milieu shifts. Although activating neutrophils represents their primary and more extensively studied function, RTECs also exhibit direct neutrophil-recruiting capacity. Ferreira et al. leveraged spatial transcriptomics, single-cell sequencing, and CODEX imaging to demonstrate that in renal IRI models, neutrophils specifically infiltrate the outer cortex at the corticomedullary junction via chemokine Atf3 secreted by S3 proximal tubule subpopulations. In contrast, cecal ligation and puncture (CLP)-induced AKI models showed minimal neutrophil recruitment at this site (21, 31). Correspondingly, whereas neutrophil migration inhibition significantly attenuated kidney injury in IRI mice, no comparable protection occurred in CLP-induced AKI (34).

Additionally, our prior single-cell sequencing analysis of murine post-IRI renal tissue revealed neutrophil-specific upregulation of CXCR2 and interleukin-1β (IL-1β) within this microenvironment. Neutrophils likely engage in recruitment and migration through CXCR2 interactions with C-X-C motif chemokine ligand 1 (CXCL1) secreted by injured proximal tubules (PTs) (22). Notably,CXCL1, the murine homolog of human CXCL8/Interleukin-8 (IL-8), is significantly upregulated in renal tissue post-IRI. Bioinformatic analysis further indicates that human CXCL8 participates in NETosis and inflammatory responses during IRI associated delayed graft function (DGF) (32). Pharmacological inhibition of CXCL1/CXCL8 substantially reduces NET formation, attenuates tubular necrosis and inflammation, and partially improves renal function (32). Previous literature has confirmed that the interaction of SPP1 with various integrins (alpha v [αv] integrins (33), Integrin β1 (34), Integrin β3 (35), etc.) and CD44 (36) promotes cell adhesion and migration. Consistent with these findings, our sequencing results revealed that both the SPP1-CD44 and MIF-CD74/CD44/CXCR2/CXCR4 ligand-receptor pairs exhibited strong signaling activity, indicative of their role in mediating interactions between RTECs and neutrophils (22). This finding is consistent with a previous study by Jinhong Li et al., which showed that in cisplatin-induced acute kidney injury, loss of RTEC-derived MIF suppressed the infiltration of inflammatory cells such as neutrophils into renal tissue, an effect likely mediated by the CD74 receptor (37). Further to chemokine pathways, proximal tubular epithelial cells (PTECs) post-AKI upregulate the neutrophil adhesion receptor DPEP1, exhibiting expression akin to peritubular capillaries. DPEP1 is therefore established as another key mediator of tubular-driven neutrophil recruitment (27).

Thus, during the early phase of AKI, injured PTs contribute to neutrophil recruitment and migration toward sites of injury through secretion of chemokines and expression of adhesion receptors. Unlike vascular endothelial cells, which primarily mediate neutrophil extravasation from circulation, renal tubular cells guide intratissular neutrophil trafficking to precise injury locations. Furthermore, PTs activate neutrophils to amplify local inflammation.

### 3.3 Recruitment of neutrophils by leukocytes

During early AKI, activated macrophages recruit neutrophils through diverse mechanisms to drive inflammation. In both renal IRI and folic acid-induced AKI (FA-AKI) models, ferroptotic resident renal cells recruit and activate macrophages rather than directly recruiting neutrophils. These activated macrophages upregulate surface CXCL1/C-X-C motif chemokine ligand 2 (CXCL2), thereby mediating neutrophil recruitment and inflammatory responses (38). In lipopolysaccharide (LPS)-induced murine AKI models, single-cell RNA sequencing of renal tissue identified a novel pro-inflammatory CCL5+ macrophage subset. This population potentially recruits neutrophils via the CCL5-CCR1 chemokine axis, mediated by C-C motif chemokine ligand 5 (CCL5) binding to C-C motif chemokine receptor 1 (CCR1) (39). However, monocyte-derived macrophages exhibit significant functional heterogeneity across subsets, leading to paradoxical roles in neutrophil recruitment. Unlike CX3CR1^low^ Ly6C^high^ monocytes, which are recruited and further differentiate into pro-inflammatory cells, circulating CX3CR1^high^ Ly6C^low^ monocytes are recognized as patrolling monocytes. These cells crawl along endothelial surfaces, maintaining endothelial integrity (40). Based on this evidence, researchers identified a population of CX3CR1^high^ Ly6C^low^ CD169^+^ cells in the kidney. These cells were categorized as vascular-resident monocytes/macrophages that suppress endothelial hyperactivation. By inhibiting endothelial ICAM-1 expression, they limit excessive neutrophil infiltration, thereby preventing exacerbation of renal IRI (40). This evidence suggests that monocyte-macrophage regulation of neutrophils may be associated with their phenotypic complexity. Other leukocytes (e.g., T cells, neutrophils themselves) may enhance neutrophil recruitment indirectly by secreting cytokines or granule proteins that amplify adhesion or chemotaxis in resident cells. Nevertheless, evidence for such mechanisms in AKI remains limited, and no systematic understanding has yet emerged.

## 4 Delayed neutrophil death drives NETosis

Under physiological conditions, mature neutrophils have a short lifespan of hours to days, they undergo spontaneous apoptosis that is mediated by Bcl-2 protein family-regulated mitochondrial outer membrane permeabilization along with caspase-dependent DNA fragmentation, and are subsequently cleared (41–43). This transient nature maintains neutrophil homeostasis and prevents host tissue damage (44). At inflammatory sites, recruited neutrophils achieve a several-fold lifespan extension via stimulation by IFN-γ, LPS, pathogens, or adhesion molecules, which activate the PI3K-Akt/NF-κB and ERK1/2-MAPK pathways to sustain anti-apoptotic proteins (e.g., Myeloid cell leukemia 1(Mcl-1)) (45–48). Consequently, this disrupts neutrophil homeostasis in both tissues and circulation, creating pathogenic implications for organ damage.

Macrophage phagocytosis serves as the primary mechanism for terminal neutrophil clearance. Normally, dying neutrophils emit “clearance signals” by releasing diffusible mediators such as DAMPs, which recruit macrophages to the site of cell death. Surface-exposed phosphatidylserine and other ligands then engage macrophage receptors (e.g., integrins, scavenger receptors) via bridging molecules, facilitating phagocytic removal of apoptotic neutrophils (49). Delayed clearance may result from impaired emission of these neutrophil death signals. Recent research demonstrates that early EGFR activation during IRI sustains Mcl-1 expression, whereas myeloid-specific EGFR knockout promotes renal neutrophil apoptosis and enhances macrophage efferocytic capacity. This cascade accelerates early neutrophil clearance, ultimately facilitating repair of IRI-AKI and attenuating fibrosis (50).

NETosis (specifically referring to suicidal NETosis, classification detailed in Section 4.2), is considered a unique neutrophil death modality. Intriguingly, delayed neutrophil apoptosis has been shown to promote NET formation in multiple inflammatory diseases (51, 52). Subsequent mechanistic studies demonstrated that apoptosis delay prolongs apoptotic signaling-activated Gasdermin E (GSDME) pores, driving calcium-dependent peptidylarginine deiminase 4 (PAD4) activation and chromatin decondensation to permit NETosis initiation in neutrophils after apoptosis (45). Moreover, stimulation signals received by neutrophils may overlap with death signals from other pathways (e.g., necrosis, pyroptosis), thus NETosis is recognized as a secondary death modality for inadequately cleared neutrophils (53, 54). This phenomenon likely constitutes the fundamental mechanism of NET formation in inflammatory milieus and implies that NETosis can be superseded by alternative neutrophil death pathways. For instance, mesenchymal stem cell (MSC)-derived apoptotic vesicles (apoVs) shift neutrophil death from NETosis to apoptosis via apoV-Fas ligand-mediated Fas activation, thereby ameliorating sepsis-induced multi-organ dysfunction (including renal impairment) in mice (55).

## 5 Formation pathways, classification, and functions of NET

### 5.1 Pathways of NET formation

NET formation can be categorized into two main pathways based on dependence on NADPH oxidase (NOX), which involve signal transduction through the activation of distinct key enzymes. The selection of the NET formation pathway is primarily influenced by the initial activating factors for neutrophils in the microenvironment (e.g., platelets, microorganisms, LPS, immune complexes). Different environmental stimuli activate different NETosis pathways. The NOX-dependent pathway is characterized by ROS generation (56). Activated ROS within neutrophils inhibit actin polymerization, promoting the release of proteases such as myeloperoxidase (MPO) and neutrophil elastase (NE). These enzymes degrade histones and structural proteins (e.g., Gasdermin D, GSDMD), compromising membrane stability and increasing intracellular calcium ion concentration (57–59). Notably, MPO not only kills pathogens by catalyzing hypochlorous acid (HOCl) but also directly activates NE. Together, they play a core regulatory role in NOX-dependent NET formation (57, 59).

Conversely, in the NOX-independent pathway, the absence of significant ROS generation and subsequent protease-mediated histone cleavage means NET formation relies more heavily on the downstream enzyme PAD4. Under the regulation of intracellular Ca²^+^ concentration, PAD4 catalyzes histone citrullination (converting positively charged arginine residues to uncharged citrulline), weakening histone-DNA binding and promoting chromatin decondensation (60). PAD4 is a classic molecule in the NET formation pathway. It was found to be highly induced in leukocytes 24 hours after renal ischemia, coinciding with histone H3 citrullination and NET formation in the kidneys (61). Further research revealed that PAD4 in renal proximal tubules appears to promote IRI-AKI by facilitating tubular epithelial cell apoptosis, while bone marrow-derived PAD4 preferentially contributes to promoting renal neutrophil infiltration and inflammation following renal I/R (62).

Additionally, intracellular Gram-negative LPS triggers the assembly of the non-canonical inflammasome, leading to caspase-4/11 activation and cleavage of GSDMD into its p30 fragment (63). This pathway, which is similarly independent of NADPH oxidase 2 (NOX2), is specifically termed non-canonical NETosis. This Caspase-11/GSDMD-dependent NET formation can be induced by uric acid. It mediates inflammation and renal fibrosis during the progression of hyperuricemic nephropathy by enhancing the production of α-smooth muscle actin (α-SMA) in macrophages (64). However, its role in AKI has not yet been reported.

### 5.2 Relationship between NETosis types and their dualistic functionality

NETosis does not invariably result in neutrophil death. Depending on the pathogen type and tissue microenvironment, it can manifest in four distinct forms: suicidal NETosis, vital NETosis, noncanonical NETosis, and mitochondrial NETosis (65). Suicidal and mitochondrial NETosis rely on ROS activation, whereas vital and noncanonical NETosis occur independently of ROS (66, 67). Notably, suicidal NET formation requires several hours, whereas ROS-independent vital NET formation can occur within approximately 30 minutes. In both suicidal and noncanonical NETosis, NET release coincides with cellular rupture. Neutrophils undergoing these forms lose their capacity for phagocytosis, crawling, and other multifunctional activities. Conversely, vital NETosis occurs independently of lytic cell death. Neutrophils expel NETs via vesicular release without nuclear membrane rupture and retain their functional capabilities thereafter (56, 68).

Studies comparing IRI-AKI and sepsis-associated AKI (SA-AKI) revealed distinct neutrophil phenotypes. Neutrophils recruited in IRI-AKI are characterized by lower levels of CD11b, CD54, and CD95, whereas neutrophils in CLP-induced SA-AKI predominantly exhibit a CD11b^high^, CD54^high^, and CD95^high^ phenotype, indicating enhanced phagocytic capacity, ROS generation, rolling, and adhesion functions in the latter¹^9^. Furthermore, a higher proportion of Sytox-positive cells was observed under IRI conditions¹^9^, suggesting a greater propensity for NETosis in IRI-AKI, with evidence pointing specifically toward suicidal NETosis. Critically, however, current literature indicates that the functional roles of NETs in IRI-AKI and SA-AKI are not entirely consistent (see Section 6 for details). This disparity suggests that tissue-specific environmental signals and the specific type of NETosis occurring may be key determinants of NET functionality.

The critical mechanism underlying the double-edged sword effect of NETs is generally attributed to an imbalance between their formation and clearance. During inflammation, neutrophils eliminate pathogens via NETs; yet, the non-specific components of excessively released NETs exert pro-inflammatory and damaging effects within tissues. Notably, although NETs were initially thought to kill entrapped bacteria through their associated antimicrobial proteins, subsequent research revealed that effective bacterial killing requires not only NET-embedded MPO but also hydrogen peroxide (H_2_O_2_) supplied by viable neutrophils (69–71). Consequently, while different NET types exhibit pathogen containment capabilities, suicidal NETosis proves comparatively less effective in pathogen eradication. By contrast, vital NETosis, which preserves neutrophil viability, facilitates more efficient pathogen clearance. In the bloodstream context, a primary function of NETs is to contain pathogens and prevent dissemination. Nevertheless, complexes formed by NETs entrapping pathogens, platelets, and cellular debris readily initiate immunothrombosis (72). Excessive immunothrombosis may progress to vascular occlusion and subsequent multi-organ failure (73–75). This pathological process contributes to diverse AKI types and related conditions, including IRI (76), sepsis (77), *Escherichia coli* infection (78), and SARS-CoV-2 infection (COVID-19) (79). In this context, we further focus on the potential contribution of non-specific NET components to the hypercoagulable state observed in disease. This includes histone-induced platelet activation (80), as well as DNA- and serine protease-mediated activation of factor XII and tissue factor, respectively (81, 82). However, despite these detrimental effects mediated by non-specific NET components, distinctions between different NET types remain unexplored. Variations in their release kinetics and mechanisms may confer differing degrees of cytotoxicity. Collectively, these mechanisms may partially explain the differential impact of NETs on inflammatory regulation across distinct types of AKI.

## 6 Receptors and signaling pathways in NET formation under AKI conditions

**Figure 1:**
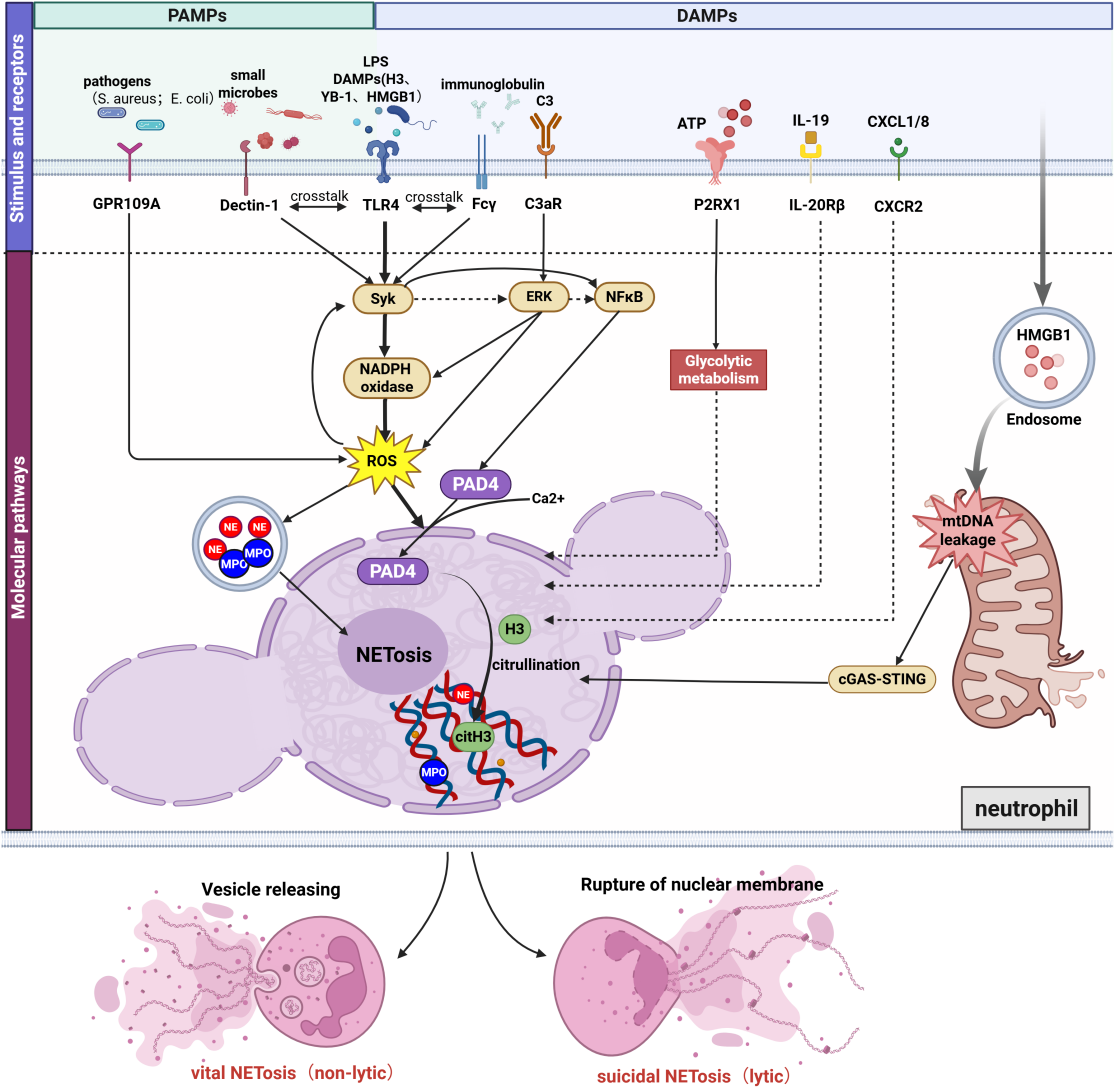
Mechanisms of NET formation induced by diverse molecular patterns in AKI. NET formation in AKI occurs through: (1) Bacterial-Induced GPR109A Modulates NETosis by Regulating the ROS/PAD4/CitH3 Signaling Axis; (2) DAMPs (histones, YB-1,HMGB1,etc) and LPS promote NETosis via TLR4, with TLR4 cross-talking with Dectin-1 and Fcγ receptors through activation of the shared downstream pathway Syk/NFκB/PAD4; (3) C3a binding to neutrophil C3aR activating the ERK/ROS/PAD4 pathway; (4) P2RX1 activation by ATP driving NETosis via enhanced glycolysis; (5) IL-19-IL-20Rβ and CXCL1/8-CXCR2-mediated NETosis (molecular mechanisms undefined); (6) Endosomal HMGB1 triggering mitochondrial DNA leakage and cGAS/STING-dependent NETosis. Besides, ROS activation promotes the release of proteases (MPO and NE). Ca²^+^-mediated PAD4 activation promoting histone citrullination and chromatin decondensation. The final forms of NET formation are divided into lytic and non-lytic types. Figure created with BioRender.com.

The process of NET formation typically relies on the specific binding of ligands to transmembrane receptors or specialized signaling transmission mechanisms such as endosomes, which subsequently trigger the cascade activation of downstream signaling pathways. In AKI, NET induction may originate from stimuli such as pathogen-associated molecular patterns (PAMPs) or damage-associated molecular patterns (DAMPs). These stimuli can coexist across different pathological contexts, yet the signaling profiles vary depending on the specific pathological environment. Consequently, the signaling pathways involved in NET formation exhibit certain mechanistic commonalities but also distinct differences across various pathological subtypes of acute kidney injury. Below, based on a summary of different categories of stimulus molecules and their corresponding receptors, we illustrate the network of pathways promoting NET formation in AKI in **Figure 1**. The key neutrophil molecules validated in AKI animal models to mediate NET formation, along with major findings, are summarized in **Table 1**.

**Table 1:** Validated key molecules and mechanisms mediating NET formation in AKI models.

Mechanism types	Disease models	Key molecules	Key findings	Refs.
Mediator-membrane receptor-mediated NET formation	Renal I/R LPS-induced AKI mouse model	YB-1/DNA complex, TLR4	The YB-1/DNA complex activates TLR4 to promote NET formation.	(11)
	Renal I/R mouse model	Human CXCL8 (mouse CXCL1) (receptor not mentioned)	Inhibiting the expression and function of CXCL8/CXCL1 reduces NETs production.	(32)
	Renal I/R mouse model	Histone、TLR4/9	Histones released from necrotic cells can induce NET formation via TLR4/9.	(84)
	Renal I/R mouse model	C3a、C3aR	Binding of C3a to C3aR on the neutrophil envelope activates neutrophils and induces NET release via the ERK/ROS/PAD4 pathway.	(93)
	Renal I/R mouse model	Extracellular ATP、 P2RX1	Activation of P2RX1 promotes platelet ATP release, which subsequently enhances neutrophil glycolytic metabolism and NET formation through P2RX1 activation.	(98)
	Renal I/R mouse model	Extracellular DNA (receptor not mentioned)	Necrotic RTECs release extracellular DNA, which in turn activates platelets, leading to platelet-neutrophil interactions and NET formation.	(76)
	AAN mouse model	IL-19、IL-20Rβ	Damaged RTECs release IL-19, which mediates NET formation via IL-20Rβ expression on neutrophils.	(107)
	Oxalate-induced AKI mouse model.	HMGB1(receptor not mentioned)	HMGB1 promotes NINJ1-dependent NET formation.	(85)
	AKI induced by mechanical ventilation in mice.	HMGB1 (receptor not mentioned)	Mechanical ventilation mediates the HMGB1-NETs signaling pathway.	(10)
	Sepsis mouse model (CLP) (kidney tissue)	HMGB1 in exosomal form	HMGB1 triggers mitochondrial DNA leakage in neutrophils and induces NET formation by activating the cGAS/STING pathway.	(104)
	Sepsis mouse model (CLP) (kidney tissue)	GPR109A	GPR109A controls NET formation by regulating the ROS/PAD4/CitH3 signaling axis.	(108)
Key molecules within neutrophils mediate NET formation.	Renal I/R mouse model	PAD4	PAD4 in neutrophils mediates renal NET formation.	(61)
	A mouse model of lupus nephritis induced by Fcgr2b-/- was used to establish an IRI-induced AKI model.	Spleen tyrosine kinase, Syk, NF-kB	*In vitro*: Inhibition of Syk and NF-κB attenuates PMA- and LPS-induced NETosis. *In vivo*: Syk inhibitor alleviates NETosis and glomerular apoptosis.	(90)

### 6.1 TLR4/Syk/NFκB/PAD4 pathway

Toll-like receptors (TLRs) constitute a ubiquitous class of pattern recognition receptors (PRRs) that detect DAMPs and PAMPs within tissues. Human neutrophils express all TLRs except Toll-like receptor-3(TLR3) on their membrane, and several TLRs have been implicated in mediating NET formation (83). Within AKI, Toll-like receptor-4(TLR4) has been identified as key receptor for NET induction stimulated by extracellular histones (84), Y-box binding protein 1(YB-1) (11),high-mobility group box 1 (HMGB1) (10, 85, 86), and other activating proteins.

Recent research highlights that TLRs on the neutrophil surface engage in crosstalk with various other receptors, collectively influencing NET formation in AKI. C-type lectin-like receptors (CLRs) represent one such family, with Dectin-1 and Dectin-2 both reported to participate in NET formation (87, 88). In a mouse model of IRI-AKI with pre-operative oral *Candida albicans* administration, TLR4 activation by LPS/DAMPs and Dectin-1 stimulation by the fungal (1→3)-β-glucan converged on the common Syk/NF-κB/PAD4 signaling axis to synergistically induce NET formation, thereby inhibiting *C. albicans* dissemination (89). Similarly, in a lupus mouse model of IRI-AKI, it was found that Syk can significantly increase dsDNA levels by enhancing neutrophil apoptosis and NETosis, thereby exacerbating lupus activity (90). This effect was further amplified by Fcgr2b⁻/⁻, potentially through activation of the TLR-4/Syk signaling axis, suggesting possible crosstalk between FcgRs and TLR-4 that warrants further investigation (90). Syk inhibitors have been shown to reduce NETosis *in vivo* and *in vitro (* 90), suggesting a viable therapeutic target for future translation.

### 6.2 C3aR/ERK/ROS/PAD4 pathway

Neutrophils express a variety of complement receptors that have been demonstrated both *in vivo* and *in vitro* to mediate NETosis. Our group’s previous single-cell RNA sequencing data revealed that upregulated C3 participates in the interaction between injured RTECs and CXCR2-expressing neutrophils via the C3-C3aR1 axis. (91). Consistent with our findings, complement C3 upregulation after IRI has been shown to drive renal injury, as evidenced by attenuated pathology and reduced neutrophil infiltration/NETosis in C3-KO mice, while *in vitro* validation demonstrates C3a-induced NET formation through CitH3 and MPO co-localization in extracellular DNA structures (92). Furthermore, C3aR-deficient (C3aR-/-) mice were generated to knockdown the C3a receptor, and it was demonstrated that the C3a-C3aR axis further contributes to IRI-AKI through the ERK/ROS/PAD4/NETosis pathway (93), highlighting C3a-C3aR1 as a highly promising therapeutic target for future NETosis-directed therapies. Notably, another complement component, C5a, can promote the expression of anti-neutrophil cytoplasmic autoantibody (ANCA) antigens on neutrophil membranes via the C5a receptor (C5aR), thereby amplifying ANCA-mediated neutrophil activation and inducing necrotizing crescentic glomerulonephritis (94, 95). However, whether the C5a-C5aR axis is involved in mediating NET formation requires further investigation.

### 6.3 P2RX1-glycolytic pathway

Purinergic receptors are activated by extracellular nucleotides such as ATP and their metabolic end product adenosine (ADO), with ADO inhibiting NET formation via P1 receptors (96, 97), while ATP promotes NETosis through P2 receptor signaling. In IRI-AKI, platelet P2RX1-mediated glycolytic metabolism supports extracellular ATP release, which subsequently activates neutrophil P2RX1 to drive their glycolytic flux and NET formation (98). Separate studies have demonstrated that extracellular ATP binding to ionotropic P2X receptors (P2XR) opens the receptor channels to promote Ca²^+^ influx, which subsequently activates either the p38 MAPK pathway or phospholipase A2 (PLA2R) signaling, thereby facilitating bacterial killing and triggering inflammatory responses (99). However, whether P2XR activation promotes neutrophil-mediated NETosis via the aforementioned signaling pathways remains unreported, presenting a potential direction for future research.

### 6.4 mtDNA-cGAS-STING pathway

Activated by binding endogenous DNA (mitochondrial DNA [mtDNA]/nuclear DNA) or bacterial DNA, cytosolic DNA sensor cGAS catalyzes second messenger cGAMP synthesis to stimulate STING-dependent inflammatory signaling (100, 101). In cisplatin-induced AKI, this pathway critically mediates cisplatin-triggered mtDNA leakage into tubular cytosol through BCL-2-like protein 4 (BAX) pores, activating the cGAS-STING/TBK1/NF-κB p65 axis that exacerbates post-injury inflammation in proximal tubular cells, whereas STING knockout attenuates neutrophil migration and ameliorates tubular inflammation (102). Consistent with these findings, *in vitro* experiments demonstrated that hypoxia/reoxygenation (H/R) treatment of neutrophils induces mitochondrial dysfunction and subsequent mtDNA release into the cytosol, where the leaked mtDNA triggers NETosis via the cGAS–STING pathway (103). Similarly, a cascade involving HMGB1 lactylation-mediated NETosis through the mtDNA-cGAS-STING pathway has been identified in murine SA-AKI, where elevated blood lactate initiates H3K18 lactylation in macrophages, leading to HMGB1 lactylation and secretion, which in turn induces mtDNA leakage in neutrophils and activates cGAS-STING to promote NET formation (104, 105). However, it remains unclear whether mtDNA serves as the sole or primary DNA source for cGAS activation, and how the cGAS–STING pathway contributes to chromatin decondensation and NETosis. The regulatory relationship between this pathway and known key effector molecules, such as NE, MPO, and PAD4, warrants further investigation.

### 6.5 Additional pathways

Previous *in vitro* studies revealed that crosstalk between the FcγRIII engagement-induced Syk-ERK pathway and the PMA-induced PKC signaling pathway enhances NET formation in mature granulocytes (dHL-60) by boosting ROS production and the generation of pro-inflammatory cytokines IL-8 and TNF-α (106). Research using both *in vivo* CLP-induced AKI models and *in vitro* studies with a variety of stimuli, including *Staphylococcus aureus* (*S. aureus*), has shown that GPR109A promotes NET formation via the ROS/PAD4/CitH3 axis (108). However, other research reports ROS-independent NETosis during *S. aureus* infection, highlighting a mechanistic heterogeneity that poses a challenge for identifying common intervention targets (109). Furthermore, in aristolochic acid-induced AKI, injured RTECs release interleukin-19 (IL-19) which promotes NET formation via neutrophil-expressed IL-20 receptor beta (IL-20Rβ) (107). Besides, Irf8 deficiency enhances neutrophil recruitment and NET formation post-AKI (110). However, current understanding of these mechanisms remains incomplete and requires further investigation.

## 7 The role of NETs in AKI induced by diverse etiologies

**Figure 2:**
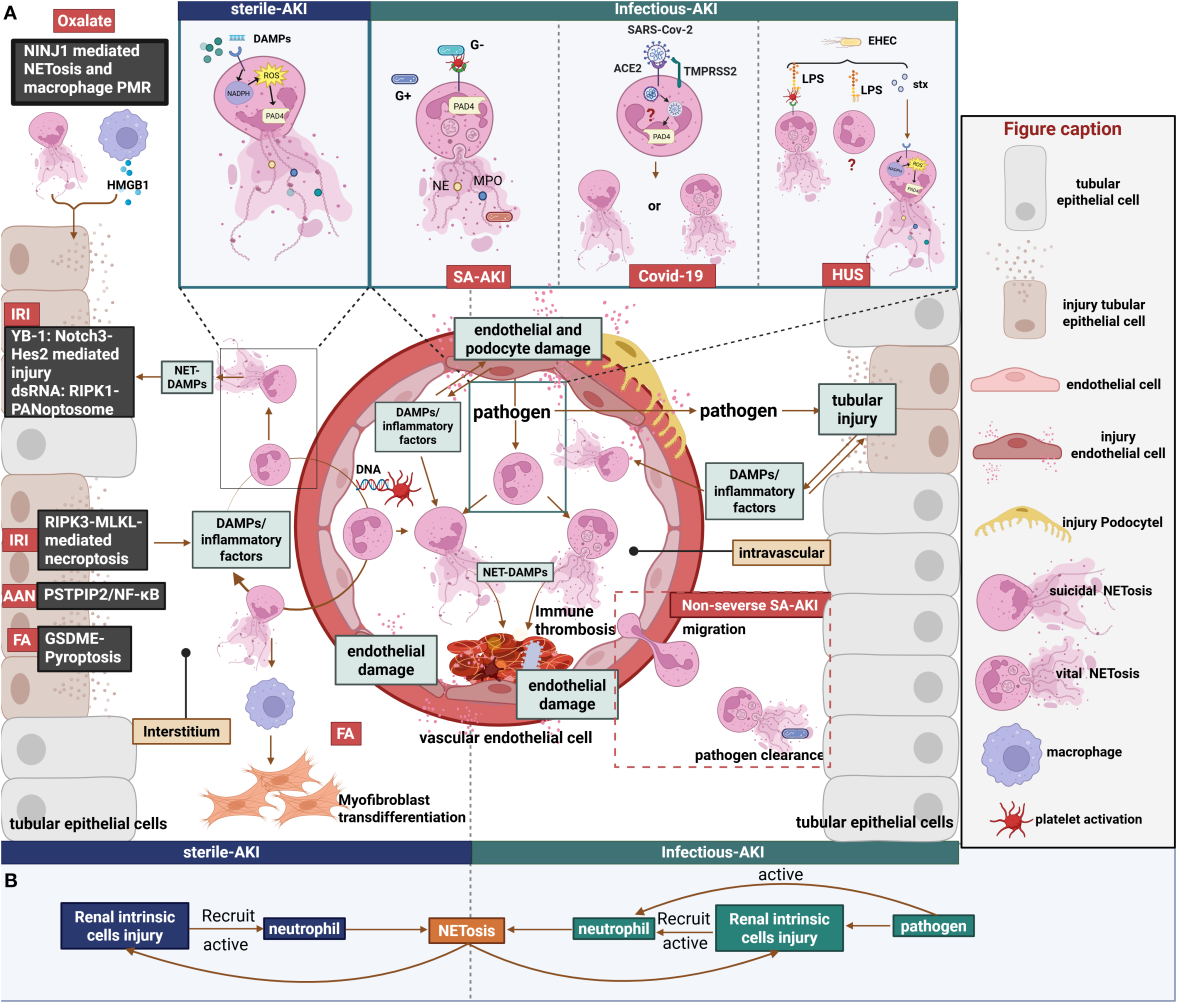
Role of NETs in sterile and infectious AKI. **(A)**1. Left panel: Renal IRI directly induces RIPK3-MLKL-dependent necroptosis in renal tubules, triggering the release of DAMPs and inflammatory cytokines. This release promotes NET formation within both the renal tissue and the vasculature. Subsequent release of NET-associated DAMPs, such as YB-1 and dsRNA, further damages RTECs and vascular endothelial cells through distinct mechanisms. Furthermore, AAN and FA-AKI damage RTECs via diverse mechanisms, thereby perpetuating a NET-associated inflammatory cycle. Notably, NETs in FA-AKI have been demonstrated to mediate macrophage transdifferentiation into myofibroblasts. Additionally, calcium oxalate crystals induce NINJ1-mediated NETosis and macrophage PMR, which synergistically promote RTEC injury. 2. Right panel: Septic AKI. Diverse forms of NET formation occur in response to various pathogens. However, as inflammation intensifies, neutrophil migratory capacity declines, leading to a predisposition for NETosis to occur predominantly within the circulation. This intravascular NETosis contributes to cytokine storm and vascular endothelial cell injury. Additionally, pathogens exert direct cytotoxic effects on renal parenchymal cells; the subsequent release of DAMPs and inflammatory mediators triggered by this damage can induce NETosis.3. Top box: Comparative mechanisms of NETosis induction. In sterile AKI, Endogenous stimuli induce suicidal NETosis via the NOX-dependent pathway. In septic AKI, platelets activated by Gram-positive bacteria or Gram-negative bacterial LPS mediate vital NETosis via a NOX-independent pathway. In COVID-19-associated AKI, the specific type of NETs generated via the SARS-CoV-2 ACE2/TMPRSS2 pathway remain unclear. In HUS, EHEC may induce different forms of NETosis mediated by Stx and LPS.**(B)**Annotated schematic diagram of NET formation and function in sterile vs. Infectious AKI settings. Figure created with BioRender.com.

The phenotype and function of neutrophils are intimately regulated by stimulatory signals within the tissue microenvironment. As a key effector product of neutrophils, NETs appear to play divergent roles in AKI of varying etiologies. Herein, we systematically review current evidence on NETs in AKI induced by multiple etiologies, with a focused analysis of their functional impacts to elucidate shared and distinct pathological contributions within different injury milieus (**Figure 2**). In particular, **Table 2** outlines the contribution of NETs to kidney tissue injury in AKI.

**Table 2:** Summary of mechanisms of NETs-induced tissue damage in AKI.

Disease model	Functional executor	Target cell	Outcome	Refs.
Renal I/R	Histone	RTECs	Necrosis	(84)
*In vivo*	dsDNA	Platelets	Activation with increased expression of platelet factor 4 (PF4)	(76)
Renal I/R	NETs (undifferentiated components)	RTECs	Apoptosis	(93)
Renal I/R、LPS-induced AKI	YB-1	Tubular cells	injury, proliferation	(11)
Renal I/R	dsRNA	Tubular cells	PANoptosis	(142)
Renal I/R mouse model	NETs (undifferentiated components)	–	Impaired mitochondrial dynamics	(98)
AAN	NETs (undifferentiated components)	Tubular cells	Apoptosis	(107)
CI-AKI	NETs (undifferentiated components)	Glomerular and peritubular capillary endothelial cells (RTECs)?	Apoptosis, pyroptosis	(149)
FA-induced AKI-to-CKD	NETs (undifferentiated components)	Macrophages	Myofibroblast transdifferentiation	(148)

### 7.1 Infection-associated AKI

In infection-associated AKI, circulating neutrophils are activated by various stimuli, including direct contact with invading pathogens and endogenous signals released from damaged local tissues. Unlike sterile inflammation, infection-induced NETosis exhibits a far greater dependence on the specific nature of the invading pathogen, significantly complicating the role of NETs in this setting. Furthermore, because the bloodstream serves as a primary route for pathogen dissemination, the pathological consequences of infection-related NETs are predominantly centered on damage to the vascular endothelium.

#### 7.1.1 Sepsis-associated acute kidney injury

SA-AKI is a prevalent form of AKI in humans, with hemodynamic alterations stemming from renal microcirculatory dysfunction serving as the central mechanism underlying its pathogenesis (111, 112). Microcirculatory abnormalities prolong neutrophil retention within peritubular capillaries, which facilitates the sustained release of inflammatory mediators, exacerbating local inflammation and thereby driving the development of septic AKI, while neutrophil infiltration into the renal parenchyma appears not to be a major factor in this process (21, 113, 114).

The role of neutrophils and NETs in sepsis is highly context-dependent, particularly influenced by the severity of the septic environment. Based on our synthesis of SA-AKI animal studies, NETs exert a protective role only in models employing solely CLP within a 3-day observation window (104, 108), whereas they demonstrate detrimental effects when more complex modeling approaches are used (e.g., CLP with prior burn/lactate) (105, 115, 116)or when CLP observation periods are extended (104, 108) (**Table 3**). This pathogenesis likely reflects that the NET-inflammatory cascade in SA-AKI originates from circulating pathogens rather than parenchymal injury. Vital NETosis is classically triggered by core sepsis pathogens including fungal infections (117) and *S. aureus* (109), as well as by LPS-sensitized platelets (118). We hypothesize that during early-stage sepsis, neutrophils confer protection through the release of such NETs characterized by potent pathogen-capturing capacity, which effectively eliminate invading microbes. During disease progression, neutrophils with delayed apoptosis exhibit impaired vascular egress due to Toll-like receptor-2 (TLR2)-mediated CXCR2 downregulation, resulting in concurrent tissue pathogen dissemination and accelerated circulating NET formation (119, 120). Subsequently, intravascularly retained NETs exacerbate microcirculatory dysfunction through endothelial interactions, thereby inducing renal hypoperfusion, tubular necrosis, and cytokine storms (119, 121). At this stage, neutrophils receiving amplified DAMPs signals from diverse sources undergo distinct forms of NETosis that reinitiate this inflammatory cycle.

**Table 3:** Effect of NETs on animal survival and renal inflammation in sepsis.

Effect	Modeling approach	NET-Targeting groups	Observation window (post-injury)	Outcome	Refs.
protect	CLP	Dnase-/+	1-3d	NETs preserve animal survival rates.	(108)
–			4-6d	NETs exert no significant impact on animal survival rates.	
injury			7-10d	NETs decrease animal survival rates.	
–			11-16d	NETs exert no significant impact on animal survival rates.	
protect			72h	NETs exert suppressive effects on both inflammation and bacterial burdens across multiple organ systems, including the kidney.	
protect	CLP	exos-HMGB1/exos-sh-HMGB1	1-3d	NETs preserve animal survival rates.	(104)
injury			4-7d	NETs decrease animal survival rates.	
	Hemorrhagic shock followed by CLP at 24h	WT/PAD4-/-	1-14d	PAD4-/- enhances survival (days 2-14)	(116)
			24h	PAD4-/- mice exhibit superior renal functional preservation compared to WT controls.	
	Single i.p. injection of lactate (30 mg/kg) plus LPS (2 mg/kg)	—	24h	NET formation contributes to the exacerbation of AKI	(105)
	E.coli-derived LPS (2mg/kg) i.v. on post-burn day 10	Resolvin D2 -/+	Post-burn day 11(24h post-LPS challenge)	Untreated groups exhibiting elevated blood urea nitrogen levels demonstrated increased NET accumulation, evidenced by expanded NET-occupied tissue areas.	(115)

#### 7.1.2 Coronavirus infection-associated acute kidney injury

COVID-19, a major emerging infectious disease in recent years, leads to poor patient prognosis through its induction of AKI, making the pathological mechanisms underlying renal injury following SARS-CoV-2 infection a critical area requiring urgent investigation. Research by Brandon et al. demonstrated that elevated levels of cell-free DNA (cfDNA), a key component of NETs, is significantly associated with severe AKI, leading them to propose intravascular NETosis as a crucial factor in microthrombus formation and the development of COVID-19-associated AKI (79). Consistent with this, NETs were found to correlate closely with serum von Willebrand factor (vWF) levels in COVID-19-related AKI (122). This phenomenon may occur because the primary pathogenic agent of COVID-19, SARS-CoV-2, enters circulating neutrophils via binding to angiotensin-converting enzyme 2 (ACE2) and priming of the spike (S) protein by transmembrane protease serine 2 (TMPRSS2), where it replicates, activates NETosis, and releases NETs that further induce microthrombosis and vasculopathy (71, 123, 124). Subsequent studies showing that degradation of NETs by DNase I ameliorated SARS-CoV-2-induced renal injury in mice (125) support the injurious role of NETs. However, although some studies report that NETs capture and inhibit viruses like HIV-1 (126) and Chikungunya virus (127) to exert protective effects, and other literature supports dengue virus inducing vital NETosis via platelet-dependent, NOX-independent pathways (128), the specific type of NETosis directly mediated by SARS-CoV-2 remains unclear. Furthermore, SARS-CoV-2 has been found to directly induce renal tubular necrosis through signaling pathways involving TLR4, TLR3, and the interleukin-1 receptor (IL-1R) (129). Therefore, definitively determining whether NETs act as harmful or beneficial factors in this context, while also identifying the primary initiating factor in COVID-19-associated AKI, remains an unresolved and pressing question.

#### 7.1.3 Hemolytic uremic syndrome-associated acute kidney injury

Hemolytic uremic syndrome (HUS) is the most common cause of acute renal failure in the pediatric population and is etiologically associated with infection by Shiga toxin (Stx)-producing enterohemorrhagic *Escherichia coli* (EHEC), a Gram-negative bacterium. During EHEC infection, LPS and Stx represent two distinct pathogen-derived stimuli, and the presence of Stx has been shown to enhance the ability of LPS-sensitized platelets to induce NETs (130). However, studies in murine macrophages demonstrate that these stimuli activate different caspases and interact in a functionally antagonistic manner; specifically, cytosolic LPS activates caspase-11, which cleaves full-length GSDMD to generate the active pore-forming N-terminal fragment (NT-GSDMD), and subsequently, EHEC Stx-activated caspase-3 cleaves this caspase-11-generated NT-GSDMD, thereby inactivating it and consequently inhibiting pyroptosis and interleukin-1β (IL-1β) maturation (131). Nevertheless, whether Stx interferes with LPS-induced NETosis via the non-canonical pathway in neutrophils remains unexplored. Stx has long been recognized as a key pathogenic factor causing AKI in HUS. Current research reveals the role of Stx in directly inducing NET formation in HUS, where Stx stimulates the release of suicidal NETs via NOX-dependent pathways (132, 133). Subsequently, non-specific components derived from these NETs activate human glomerular endothelial cells (GEnCs), stimulating the secretion of pro-inflammatory cytokines IL-6 and IL-8, ultimately promoting microvascular inflammatory responses and thrombosis that lead to renal failure (78). Regarding the nature and role of NETs in this context, evidence further indicates that the NET degradation capacity in patients with Shiga toxin-producing *E. coli* HUS (STEC-HUS) negatively correlates with serum urea nitrogen and creatinine levels (134). Additionally, Stx can downregulate vascular endothelial growth factor A (VEGF-A) in podocytes, consequently leading to loss of glycocalyx on GEnCs, reduced binding of the complement inhibitory factor H (CFH) to GEnCs, and local activation of the complement pathway (135). Simultaneously, Stx can directly target and damage RTECs via the glycolipid globotriaosylceramide (Gb3) (136, 137). Thus, NET formation in the HUS milieu is influenced by multiple factors; although some NETs released by neutrophils effectively entrap and kill EHEC, the Stx released by EHEC and the ensuing burst of inflammatory mediators accelerate an imbalance in NET-mediated effects.

### 7.2 “Sterile” acute kidney injury

Sterile kidney injury refers to renal damage driven by immune system-mediated inflammatory responses in the absence of alloantigen stimulation, primarily encompassing ischemic renal injury and nephrotoxic renal injury (138). During the early stages of sterile AKI, NETs may exert a limited anti-inflammatory effect by entrapping necrotic tissue; however, current research indicates that compared to pathogen-targeting function, sterile-associated NETs in AKI tend to exhibit predominantly pro-injury and inflammation-amplifying properties. These detrimental effects manifest through direct cytotoxicity on epithelial and endothelial cells or through the modulation of inflammatory cytokines by influencing various immune cell populations, thereby exacerbating the tissue inflammatory cascade.

#### 7.2.1 Ischemia-reperfusion injury-induced acute kidney injury

IRI is a major cause of AKI, commonly occurring in various diseases characterized by hypoperfusion and/or hypoxia, such as renal transplantation, thrombotic diseases, sepsis, trauma, and post-cardiac surgery (139, 140). RIPK3-MLKL-dependent necroptosis in renal tubules serves as the primary driver of early renal injury post-IRI, subsequently triggering NLRP3 inflammasome activation which further accelerates necroptosis and initiates additional inflammation in a self-amplifying cycle (141). Within this context, the development of renal inflammation post-IRI is dependent on neutrophil infiltration and their direct cytotoxic mediation (21). Within this environment, endogenous DAMPs serve as crucial drivers in the NET-associated inflammatory cycle through their binding to neutrophil surface receptors, which activates intracellular signaling cascades to induce NETosis. Following NET release, non-specific molecular components of NETs induce mitochondrial dysfunction (98), cellular injury, or death (11, 84, 93, 142), ultimately forming an inflammation-amplifying cycle. In addition to histones (84) and extracellular DNA (76), the nuclear protein YB-1 has recently been identified as a novel NET component. YB-1 mediates RTECs damage and proliferation through the Notch3-Hes2 pathway, although it can be produced independently of NET release and additionally promotes NET formation (11). Furthermore, double-stranded RNA (dsRNA), another nucleic acid component, acts as a significant functional effector in NET-mediated IRI-AKI. Specifically, dsRNA released from NETs drives PANoptosis in RTECs through activation mediated by TLR3 pathway (142), although whether dsRNA itself promotes NET formation remains unelucidated.

Since the IRI milieu is primarily driven by endogenous mediators that activate neutrophils, it likely facilitates predominantly suicidal NETosis. Recent studies report that neutrophil infiltration and NOX2-dependent NADPH oxidase activation serve as major drivers in mild renal IRI but contribute minimally to moderate-severe renal IRI (15), prompting consideration of whether vital NETosis driven by NOX2-independent pathways operates within IRI contexts. Although platelets have been documented to activate neutrophils for vital NETosis (83, 118, 143), the specific NETosis phenotype induced by extracellular DNA-activated platelets in this environment remains undetermined. However, in IRI-AKI, circulating extracellular DNA-activated platelets stimulate neutrophils to release NETs that capture platelets/cellular debris and recruit immune cells for clearance, while inevitably forming immunothrombi which critically exacerbate renal injury (76). Moreover, the pathophysiological milieu of IRI-AKI in humans likely exhibits multifactorial complexity. For instance, intestinal barrier compromise may permit fungal translocation to the kidneys (89, 144), thereby creating synergistic neutrophil activation where endogenous mediators and exogenous pathogens collectively enhance NET formation while generating distinct NET subtypes. Collectively, current evidence indicates that NETs predominantly function as inflammatory cycle amplifiers within IRI milieus, and further elucidation of NETosis mechanisms and their functional impacts under these conditions will establish a robust foundation for therapeutically targeting NET regulation to mitigate disease progression.

#### 7.2.2 Other types of sterile acute kidney injury

Beyond IRI-AKI, NETs have also been identified in renal injury caused by various exogenous or endogenous nephrotoxic substances. The acute phase of aristolochic acid nephropathy (AAN), induced by the nephrotoxic component of Chinese herbs aristolochic acid, is characterized by inflammatory cell infiltration, excessive damage, and death of RTECs (145). In a murine model of aristolochic acid-induced AKI (AAI-AKI), downregulation of proline-serine-threonine phosphatase-interacting protein 2 (PSTPIP2) was identified as the critical step mediating RTEC injury and apoptosis while promoting neutrophil infiltration (107). PSTPIP2 downregulation activates the NF-κB pathway to release IL-19, which then induces NET formation via the IL-20Rβ receptor on neutrophils, and the resulting NETs subsequently cause damage to RTECs (107), thus amplifying inflammation.

Oxalate nephropathy represents a crystalline nephropathy where rapid and diffuse intranephronal crystal deposition causes substantial cellular necrosis and triggers an inflammatory cascade ultimately leading to AKI (146). Neural injury-induced protein 1 (NINJ1) plays a significant role in inducing plasma membrane rupture (PMR), and recent studies in oxalate-induced AKI demonstrate that NINJ1 oligomerization mediates both NET formation and macrophage PMR releasing HMGB1, processes which synergistically promote RTECs injury, whereas myeloid cell-targeted blockade of NINJ1 effectively attenuated kidney damage (85).

Although clinically rare, FA-AKI is frequently used in experimental animal models and is pathologically characterized by extensive tubular necrosis, loss of brush borders, and significant mitochondrial reduction (147), with its mechanisms potentially involving dual pathways of crystal deposition and immune responses. Research found that compared to wild-type mice and mice with single knockout of either GSDMD or GSDME, double knockout of GSDMD and GSDME conferred enhanced protection against FA-AKI modeling, attenuating NET formation, macrophage polarization, and subsequent renal fibrosis development (148), an effect likely attributable to the concurrent blockade of GSDME-mediated RTECs pyroptosis within the inflammatory milieu together with GSDMD-mediated NET release and the subsequent NET-driven macrophage-to-myofibroblast transition (MMT).

Finally, a study first identified NETs in renal tissues of mice with contrast-induced AKI (CI-AKI), with NETs primarily aggregating within glomeruli and peritubular capillaries, and subsequent inhibition of NET production through either degradation of extracellular DNA or PAD4 blockade alleviated both apoptosis and pyroptosis in CI-AKI kidneys (149). Collectively these data indicate that NETs predominantly play a detrimental role in these AKI settings similar to IRI-AKI, although the specific NET species involved and their underlying pathological mechanisms remain poorly characterized and merit further exploration.

### 7.3 Summary

In summary, the pathogenesis of AKI is complex and broadly categorized into sterile and infectious types, centered on the interplay between inflammatory responses and cellular injury. In sterile AKI, resident renal cells (primarily tubular epithelial and endothelial cells) serve as the principal targets. These injured cells release chemokines and cytokines that recruit neutrophils to the injury site. Within the tubulointerstitial compartment, neutrophils undergo further activation and NETosis, amplifying local inflammation. Conversely, in infectious AKI (particularly SA-AKI), intravascular pathogens directly activate neutrophils, prompting their migration to multiple organs. This process triggers systemic inflammatory response syndrome, culminating in multi-organ dysfunction. Critically, both injury modes establish a feed-forward amplification loop between neutrophils and resident renal cells, markedly exacerbating inflammatory cascades and renal damage.

## 8 Targeting NETs for therapeutic intervention

**Figure 3:**
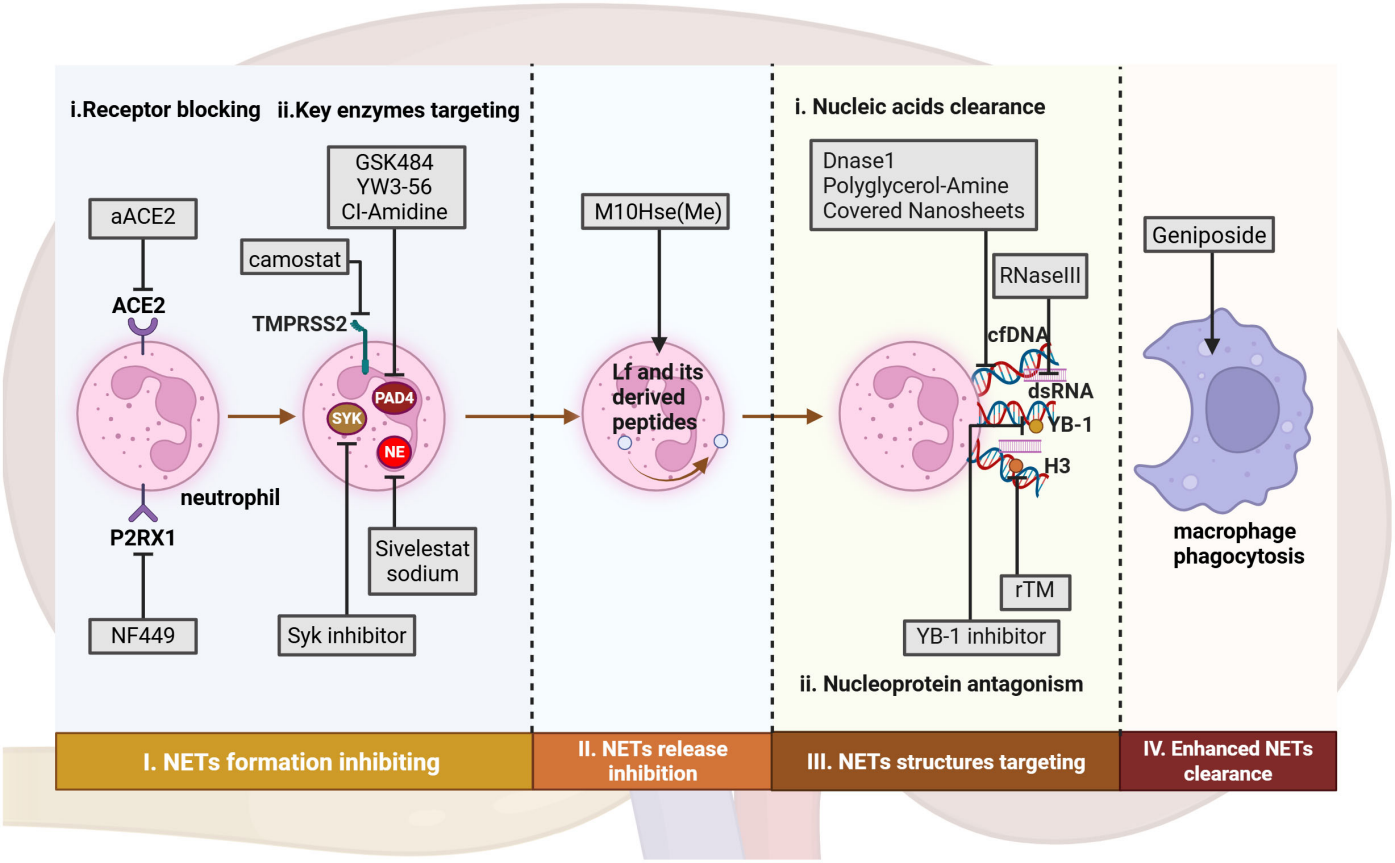
Therapeutic approaches targeting NETs from formation to clearance in AKI. In AKI, potential therapeutic approaches include: (I) Targeting NET formation by inhibiting ligand-receptor interactions and key enzymes involved in neutrophil activation; (II) Inhibiting NET release through interference with electrostatic interactions. (III) Targeting NET components via non-specific degradation of nucleic acids and antagonism of histones. (IV) Promoting NET clearance by enhancing macrophage phagocytic capacity. aACE, anti-hACE2 antibody. rTM, recombinant thrombomodulin. Figure created with BioRender.com.

As the formation mechanisms and functional roles of NETs in the pathological processes of AKI are increasingly elucidated, therapeutic agents targeting key points in the NET formation pathway and their critical pro-inflammatory components have emerged as promising novel drugs with significant clinical potential. This section will outline current and potential therapeutic strategies aimed at blocking NET formation, antagonizing essential pro-inflammatory structures within NETs, and directly clearing existing NETs (**Figure 3**, **Table 4**).

**Table 4:** Advances in NETs-targeted therapies in AKI.

Intervention/Drug	Target	Model	Outcome	levels of evidence	Refs.
NET formation inhibiting					
NF449	P2RX1	I/R-AKI	Decreased NET formation; Improved mitochondrial function; Ameliorated IRI	animal models	(98)
GSK484	PAD4	I/R-AKI	Attenuated lung injury after renal I/R; Alleviated systemic inflammation	animal models	(9)
		Cancer-associated AKI	Improved renal function	animal models	(151)
		CI-AKI	Decreased NET formation; Improved renal function; Attenuated injury to glomerular and peritubular capillary endothelial cells; Decreased renal cell apoptosis and pyroptosis (particularly in renal tubular epithelial cells)	animal models	(149)
YW3-56		I/R-AKI	Alleviated renal injury.	animal models	(61)
Cl-amidine	PAD	I/R-AKI	Decreased NET formation; Alleviated renal injury.	animal models	(84)
		LPS-AKI	Alleviated renal injury; Improved renal function; Increased survival rate.	animal models	(152)
Sivelestat sodium	NE	IRI-AKI	Decreased NET formation; Alleviated renal injury.	animal models	(150)
Syk inhibitor	Syk	IRI-AKI	Decreased NET formation; Alleviated renal injury.	animal models	(89, 90)
NET release inhibition					
M10Hse (Me)	/	IRI-AKI	Decreased NET formation; Alleviated renal injury; Improved renal function; Increased survival rate.	animal models	(154)
NET structures targeting					
Dnase1	cfDNA	CI-AKI	Same as above (results of GSK484 in CI-AKI).	animal models	(149)
Polyglycerol-Amine Covered Nanosheets	cfDNA	IRI-AKI、LPS-AKI	Reduced serum cfDNA levels; Improved renal function; Alleviated renal injury.	animal models	(158)
		AKI patients		*In vivo* (Human serum)	
RNaseIII	dsRNA	OGD/R	Alleviated RTECs PANoptosis.	*In vivo*	(142)
rTM	Histone	IRI-AKI	Decreased in histones, HMGB1, and NET in the lungs; Reduced renal and pulmonary vascular permeability; Alleviated pulmonary inflammation (only IL-6 decreased in the kidneys).	animal models	(160)
		SA-AKI	Decreased renal medullary NET; No improvement in renal function.	animal models	(161)
YB-1 antibody	YB-1	IRI-AKI、LPS-AKI	Decreased NET formation; Alleviated renal injury.	animal models	(11)
Enhanced NET clearance					
Geniposide	Macrophages	Ventilator-associated AKI	Enhanced macrophage phagocytosis; Decreased NET formation.	animal models	(10)

### 8.1 Targeting NET formation

Inhibiting key receptors on neutrophil membranes or critical enzymes within neutrophil signaling pathways can block the transmission of stimulatory signals thereby suppressing NET formation and preventing AKI. Research found that application of the P2RX1-specific inhibitor NF449 to IRI-AKI mice alleviated renal histological damage, serum creatinine levels, NETosis, and mitochondrial dysfunction, establishing P2RX1 targeting as an effective strategy for protecting against renal IRI (98). Furthermore, the mechanism by which SARS-CoV-2 directly induces NET formation has been progressively elucidated, with both the neutralizing anti-hACE2 antibody (aACE2) targeting the ACE2 receptor and the TMPRSS2 inhibitor camostat proven to eliminate SARS-CoV-2-induced NETs as well as reduce viral load within SARS-CoV-2-exposed neutrophils, positioning these agents as promising candidates for alleviating COVID-19-associated AKI (123). Recently, Yanqi Liu et al. validated *in vivo* the therapeutic efficacy of the neutrophil elastase inhibitor sivelestat sodium against IRI-induced renal injury through its suppression of NET formation (150). Although this study revealed the association between NETs and IRI-AKI, it did not deeply investigate the underlying pathway mechanisms. GSK484, a widely used specific PAD4 inhibitor in research, has been shown to ameliorate remote lung injury following AKI (9) and prevent cancer-associated kidney injury (151) by mitigating neutrophil infiltration and NET formation. Similarly, another specific PAD4 inhibitor YW3-56 (61) and the pan-PAD inhibitor Cl-amidine (84, 152) were proven effective in improving renal injury and enhancing survival rates in murine models of I/R-AKI and rabbit models of lipopolysaccharide-induced septic shock respectively, yet these PAD-inhibiting drugs have not undergone clinical trials, and the degree of NET inhibition or the preservation of essential NETs by such inhibitors might be critical determinants of clinical efficacy. Additionally, Syk is a confirmed key molecular target involved in NETosis within AKI, and Syk inhibitors have demonstrated NETosis-blocking activity (89, 90), suggesting that Syk inhibitors could be useful agents for preventing renal injury in patients and warranting future research attention.

### 8.2 Reducing NET release

Y-lactoferrin (Lf), an immunomodulatory and antimicrobial human neutrophil granule protein that translocates from the cytoplasm to the plasma membrane during neutrophil activation, prevents NET dissemination via charge-charge interactions and functions as an endogenous NET inhibitor (153). Studies screening various Lf-derived peptides identified the FK-12 peptide as a potent suppressor of NET formation, leading to the development of the lactoferrin-derived peptide analog M10Hse (Me), an engineered peptide that demonstrates strong NET-inhibitory effects both *in vitro* and *in vivo (* 154). Both therapeutic and preventive administration of this agent in murine models of rhabdomyolysis-induced AKI (RIAKI) exhibited significant renoprotection while completely preventing lethality, and M10Hse (Me) additionally reduced renal fibrosis occurrence during the chronic phase of AKI, indicating its potential protective role in chronic disease progression (154).

### 8.3 Counteracting NET structures

The structural components released by NETs function as crucial DAMPs within the tissue microenvironment, acting both as primary effectors exacerbating tissue damage through NETs and as key factors initiating autocrine neutrophil stimulation that amplifies inflammation. Thus, targeting these self-released NET structures represents a critical strategy for NET-focused therapies.

#### 8.3.1 Nucleic acid clearance

DNase facilitates both the depolymerization of NET-associated nucleoproteins and macrophage-mediated clearance, with animal studies demonstrating its efficacy in mitigating diverse AKI models though clinical application faces two major limitations: achieving effective drug concentrations via injection proves difficult in humans and prolonged use induces neutralizing antibodies (149, 151, 155–157). To address this challenge, novel nanomaterial-based therapies show promise. The molybdenum disulfide nanosheets coated with polyglycerol amine developed by Haiping Mao’s team efficiently clear cfDNA through charge-mediated effects, thereby inhibiting NET formation and renal inflammation, with this technology demonstrating efficacy in both murine AKI models and patient serum experiments to provide a safer and more durable therapeutic solution for NET-related diseases while exhibiting superior clinical potential compared to conventional DNase therapy (158).

Recent research demonstrates that RNase III, a dsRNA-specific endoribonuclease, enzymatically degrades dsRNA within NET structures *in vitro*. In NETs-stimulated renal tubular cells under OGD/R conditions, RNase III treatment significantly suppresses PANoptosis (142). This proposed approach of clearing NET-dsRNA to counteract NETs offers a novel method for ameliorating kidney injury though it requires further *in vivo* validation.

#### 8.3.2 Targeting nucleoprotein components

Histones constitute essential nucleoprotein components within NETs and represent significant therapeutic targets for NET-directed interventions. Recombinant thrombomodulin (rTM) exhibits the capacity to bind circulating histones thereby exerting anti-inflammatory effects (159). Building upon this foundation, research indicates that rTM treatment may inhibit remote lung injury following renal ischemia-reperfusion by blocking pulmonary histone accumulation and NET formation while failing to ameliorate I/R-induced kidney damage itself (160). Recently, Tatsuhiko Harada et al. demonstrated in a murine SA-AKI model that rTM suppresses NET-associated histones within injured kidneys predominantly in the medulla rather than the cortex, yet rTM did not improve renal function in this investigation (161). These collective findings suggest that as a histone-binding blocking agent, rTM formulations may improve clinical outcomes by inhibiting NET functionality and attenuating NET-mediated remote organ damage propagated systemically, but since rTM targets circulating histones instead of directly acting on injured tissues, it provides no significant protection against the initial insult to the primary target organ, the kidneys. Furthermore, the DNA/RNA-binding protein YB-1 has recently been classified as a NET- DAMP. Jialin Wang’s team discovered that YB-1 in IRI environments not only activates NET formation but also functions as a critical non-specific component enabling NETs to attack renal tubular cells, and through blockade of YB-1-mediated NET-related inflammatory cascades using a YB-1 antibody, they attenuated AKI progression, thereby proposing YB-1 as a novel therapeutic target for AKI (11).

### 8.4 Enhancing NET clearance

The clearance mechanisms and pathways of deceased neutrophils depend on their microenvironment and death modalities, with phagocytic immune cells primarily macrophages and other non-classical phagocytic cells capable of participating in the removal of dead neutrophils (162). Research by Pieterse E et al. revealed that endothelial cells internalize NETs through a process dependent on the receptor for advanced glycation end products and clathrin-mediated endocytosis (163). Genipin, an active component extracted from the dried ripe fruits of *Gardenia jasminoides*, was demonstrated to promote macrophage efferocytosis for NET clearance and improve AKI by activating the AMPK-PI3K/AKT signaling pathway (10). These collective findings suggest that activating endogenous NET clearance pathways may represent a promising therapeutic strategy for ameliorating AKI.

### 8.5 Clinical feasibility and risks

Currently, evidence regarding known NET intervention targets is confined to basic experimental research, and the translation and development toward clinical applications still require considerable time. Taking PAD inhibitors as an example, several compounds such as GSK484, YW3-56, and Cl-amidine have demonstrated therapeutic potential for AKI in both *in vitro* and *in vivo* studies. Further evaluation of their efficacy in large mammals may serve as a critical step toward future clinical trials. Additionally, strategies to enhance the efficacy of PAD inhibitors through improved drug delivery systems are gradually gaining attention. A controlled-release system using P (3HB) microspheres for delivering Cl-amidine has been successfully developed and optimized, which enables sustained and controllable drug release for up to 16 days and has proven effective *in vitro (* 164). It is also important to note that the specificity of drug targeting may determine its therapeutic outcome. For instance, drugs targeting the circulatory system may be more suitable for inhibiting remote organ damage (160). In summary, for NET-targeted therapy to become a viable and effective approach for AKI treatment, further research must address multiple aspects, including drug safety, specificity, and delivery mechanisms.

Moreover, due to the context-dependent dual role of NETs—highly influenced by environmental signals—targeted clearance of NETs may interfere with the body’s innate immune regulation, posing non-negligible risks for clinical translation. A key challenge that remains to be solved is how to precisely inhibit the detrimental effects of NETs without compromising their essential immune functions.

## 9 Discussion

The AKI microenvironment is a dynamic process in which neutrophils continuously receive signals from various sources during infiltration, leading to their activation and the release of NETs. On one hand, this process protects tissues from pathogens or mediators released by necrotic tissue; on the other hand, it promotes the progression and exacerbation of inflammation. To mitigate and overcome the risks and obstacles associated with NET-targeted drugs in the clinical translation of AKI treatments, researchers need to deepen their understanding of NETs. A thorough exploration of the pathophysiological mechanisms of AKI induced by different etiologies is essential to identify the balance point of NETs—considering both temporal and quantitative dimensions—and their underlying mechanisms. Based on our summary of existing evidence, the following issues warrant further investigation: (1) The differential roles of NETs across various pathological types of AKI remain inadequately characterized: ① The initial triggers of AKI induced by different factors are not well defined. ② Different types of NETosis have distinct roles and pathophysiological significance, yet the specific forms of NETosis in AKI have received little attention. (2) Non-specific components of NETs (i.e., NET-associated DAMPs) are key molecules amplifying inflammatory responses in AKI and other inflammatory environments. However, only a few specific mechanisms of NET-associated DAMPs have been reported in the context of AKI. The roles of NET components in AKI progression, as well as therapeutic strategies targeting neutrophils and NETs, require further study.

Additionally, addressing the following technical bottlenecks is crucial for advancing research in this area: (1) Difficulties in *in vitro* neutrophil studies: Neutrophils have a short lifespan, cannot be expanded *in vitro*, and are highly sensitive to environmental changes, resulting in narrow experimental windows and technical challenges that limit research depth (43, 165). (2) Unresolved heterogeneity and functional diversity of neutrophil subsets *in vivo*: Under different pathological conditions, neutrophil subsets such as low-density granulocytes (LDGs) exhibit significant functional diversity in terms of pro-inflammatory or immunosuppressive roles. However, precise classification based on genetic/proteomic profiles, origins, and functional mechanisms remains unclear, and LDGs themselves are highly heterogeneous (165–168). Modern approaches such as scRNA-seq and proteomics are trending solutions to these questions. Advances in spatial proteomics and spatial transcriptomics may further enable precise localization of neutrophils. Moreover, real-time intravital imaging techniques can significantly enhance the study of neutrophil functions in living organisms.

## 10 Conclusion

In summary, the inherent limitations of neutrophils have resulted in relatively slower research progress compared to other immune cells. Although NETs have become a research hotspot in neutrophil-related studies due to their unique structure and specific experimental methodologies, current research on AKI primarily focuses on identifying factors that induce NET formation. There remains limited clarification of the underlying cellular mechanisms, and insufficient understanding of potential variations in the types and components of NETs under different pathological conditions. Further exploration of these issues will contribute to future efforts in achieving targeted balancing of NETs in diverse disease contexts.

## Glossary

AKI: acute kidney injury
NETs: neutrophil extracellular traps
NET: neutrophil extracellular trap
RTECs: renal tubular epithelial cells
IRI: ischemia-reperfusion injury
CXCR4: C-X-C chemokine receptor type 4
CXCL12: C-X-C motif chemokine ligand 12
G-CSF: colony-stimulating factor
CXCR2: C-X-C chemokine receptor type 2
TEM: transendothelial migration
ROS: reactive oxygen species
IRI-AKI: ischemia-reperfusion injury induced AKI
ICAM-1: intercellular adhesion molecule 1
DPEP1: dipeptidase-1
HGF: hepatocyte growth factor
GAGs: endothelial glycosaminoglycans
CXCL8: C-X-C motif chemokine ligand 8
CLP: cecal ligation and puncture
IL-1β: interleukin-1β
CXCL1: C-X-C motif chemokine ligand 1
PTs: proximal tubules
IL-8: interleukin-8
DGF: delayed graft function
PTECs: proximal tubular epithelial cells
FA-AKI: folic acid-induced AKI
CXCL2: motif chemokine ligand 2
CCL5: C-C motif chemokine ligand 5
CCR1: C-C motif chemokine receptor 1
Mcl-1: myeloid cell leukemia 1
GSDME: gasdermin E
PAD4: peptidylarginine deiminase 4
MSC: mesenchymal stem cell
apoVs: apoptotic vesicles
NOX: NADPH oxidase
MPO: myeloperoxidase
NE: neutrophil elastase
GSDMD: Gasdermin D
HOCl: hypochlorous acid
NADPH oxidase 2: NOX2
α-SMA: α-smooth muscle actin
SA-AKI: sepsis-associated AKI
H_2_O_2_: hydrogen peroxide
TLRs: toll-like receptors
PRRs: pattern recognition receptors
DAMPs: damage-associated molecular patterns
PAMPs: pathogen-associated molecular patterns
TLR3: toll-like receptor-3
TLR4: toll-like receptor-4
YB-1: Y-box binding protein 1
HMGB1: high-mobility group box 1
CLRs: C-type lectin-like receptors
Syk: spleen tyrosine kinase
dsDNA: double-stranded DNA
CitH3: citrullinated histone H3
ANCA: antineutrophil cytoplasmic antibody
ADO: adenosine
PLA2R: phospholipase A2
mtDNA: mitochondrial DNA
BAX: BCL-2-like protein 4
H/R: hypoxia/reoxygenation
IL-19: interleukin-19
IL-20Rβ: IL-20 receptor beta
GPR109A: G protein-coupled receptor 109A
TLR2: toll-like receptor-2
cfDNA: cell-free DNA
vWF: von Willebrand factor
ACE2: angiotensin-converting enzyme 2
TMPRSS2: transmembrane protease serine 2
IL-1R: interleukin-1 receptor
HUS: hemolytic uremic syndrome
Stx: Shiga toxin
EHEC: enterohemorrhagic *Escherichia coli*
IL-1β: interleukin-1β
GEnCs: glomerular endothelial cells
STEC-HUS: Shiga toxin-producing *E. coli* HUS
VEGF-A: vascular endothelial growth factor A
CFH: complement inhibitory factor H
Gb3: globotriaosylceramide
dsRNA: double-stranded RNA
AAN: aristolochic acid nephropathy
AAI-AKI: aristolochic acid-induced AKI
PSTPIP2: proline-serine-threonine phosphatase-interacting protein 2
NINJ1: Neural injury-induced protein 1
PMR: plasma membrane rupture
MMT: macrophage-to-myofibroblast transition
CI-AKI: contrast-induced AKI
aACE2: anti-hACE2 antibody
Lf: Y-lactoferrin
rTM: recombinant thrombomodulin.
